# Longitudinal Interplay Between Alcohol Use, Mood, and Functioning in Bipolar Spectrum Disorders

**DOI:** 10.1001/jamanetworkopen.2024.15295

**Published:** 2024-06-07

**Authors:** Sarah H. Sperry, Audrey R. Stromberg, Victoria A. Murphy, Carly A. Lasagna, Melvin G. McInnis, Margo W. Menkes, Anastasia K. Yocum, Ivy F. Tso

**Affiliations:** 1 Department of Psychology, University of Michigan, Ann Arbor; 2 Department of Psychiatry, University of Michigan, Ann Arbor; 3 Department of Psychiatry and Behavioral Health, The Ohio State University, Columbus

## Abstract

**Importance:**

Alcohol use disorder (AUD) is present in nearly half of individuals with bipolar disorder (BD) and is associated with markedly worsening outcomes. Yet, the concurrent treatment of BD and AUD remains neglected in both research and clinical care; characterizing their dynamic interplay is crucial in improving outcomes.

**Objective:**

To characterize the longitudinal alcohol use patterns in BD and examine the temporal associations among alcohol use, mood, anxiety, and functioning over time.

**Design, Setting, and Participants:**

This cohort study selected participants and analyzed data from the Prechter Longitudinal Study of Bipolar Disorder (PLS-BD), an ongoing cohort study that recruits through psychiatric clinics, mental health centers, and community outreach events across Michigan and collects repeated phenotypic data. Participants selected for the present study were those with a diagnosis of BD type I (BDI) or type II (BDII) who had been in the study for at least 5 years. Data used were extracted from February 2006 to April 2022, and follow-up ranged from 5 to 16 years.

**Main Outcomes and Measures:**

Alcohol use was measured using the Alcohol Use Disorders Identification Test. Depression, mania or hypomania, anxiety, and functioning were measured using the 9-Item Patient Health Questionnaire, the Altman Self-Rating Mania Scale, the 7-item Generalized Anxiety Disorder assessment scale, and the Life Functioning Questionnaire, respectively.

**Results:**

A total of 584 individuals (386 females (66.1%); mean [SD] age, 40 [13.6] years) were included. These participants had a BDI (445 [76.2%]) or BDII (139 [23.8%]) diagnosis, with or without a lifetime diagnosis of AUD, and a median (IQR) follow-up of 9 (0-16) years. More problematic alcohol use was associated with worse depressive (β = 0.04; 95% credibility interval [CrI], 0.01-0.07) and manic or hypomanic symptoms (β = 0.04; 95% CrI, 0.01-0.07) as well as lower workplace functioning (β = 0.03; 95% CrI, 0.00-0.06) over the next 6 months, but increased depressive and manic or hypomanic symptoms were not associated with greater subsequent alcohol use. These latter 2 associations were more pronounced in BDII than BDI (mania or hypomania: β = 0.16 [95% CrI, 0.02-0.30]; workplace functioning: β = 0.26 [95% CrI, 0.06-0.45]). Alcohol use was not associated with anxiety over time.

**Conclusions and Relevance:**

This study found that alcohol use, regardless of diagnostic status, was associated with mood instability and poorer work functioning in BD, but increased mood symptoms were not associated with subsequent alcohol use. Given its prevalence and repercussions, dimensional and longitudinal assessment and management of alcohol use are necessary and should be integrated into research and standard treatment of BD.

## Key Points

**Question:**

How is alcohol use associated with mood and life functioning over the longitudinal course of bipolar disorder (BD)?

**Findings:**

In this cohort study of 584 adults with BD, increased alcohol use was associated with increased depression and manic or hypomanic symptoms as well as lower workplace functioning over the following 6 months, but not vice versa. Associations were more pronounced in BD type II than BD type I.

**Meaning:**

Findings of this study suggest there is an association of alcohol use with mood and work functioning, highlighting the importance of dimensional and longitudinal assessment and management of alcohol use, which should be integrated into research and standard treatment of BD.

## Teaser

This cohort study assesses the implications of alcohol consumption for depression, mania or hypomania, anxiety, and workplace functioning among adults with type I or II bipolar disorder.

## Introduction

Up to 45% of individuals with bipolar disorder (BD) have co-occurring alcohol use disorder (AUD).^1,2,3^ Although BD and AUD are associated with poor outcomes,^4,5^ their co-occurrence may further exacerbate risk.^6,7^ Yet, current treatments for BD rarely include or consider AUD management, and problematic alcohol use is often excluded in BD trials.^6^ Consequently, the research informing clinical knowledge lacks representation from nearly half of the study population, and the extent of the interplay between alcohol use and BD symptoms is largely unexplored.

Prior cross-sectional work highlights that co-occurrence of AUD and BD is associated with prolonged alcohol withdrawal, more expensive treatment, decreased functioning, increased suicidality, and increased morbidity.^1,5,8^ These findings are generally corroborated by longitudinal studies. In a 4-year longitudinal study of BD type I (BDI), a history of AUD was associated with poor recovery after mania.^8^ In a 5-year follow-up, individuals with BDI and AUD experienced more suicidal behavior and poorer social functioning compared with those without AUD.^9^ Furthermore, those with BD type II (BDII) and AUD were more likely to have a manic episode and transition to a BDI diagnosis 5 years later, highlighting that co-occurring AUD is associated with worse outcomes of BD regardless of subtype.

Reviews also highlight associations between AUD and more complex (eg, mixed or dysphoric mania^2,10^ and rapid cycling^2,10,11,12^) and severe mood symptoms,^1,6,7,13,14,15^ the onset of which can be precipitated by alcohol use.^12,16,17^ Depressive symptoms seem to both precede and occur after alcohol use in BD throughout follow-up periods ranging from 8 months^13^ to 10 years.^18^ The association of anxiety, which affects up to 45% of individuals with BD,^19^ with alcohol use in BD has been less studied. Given the bidirectional risk between AUD and anxiety,^20^ this gap in the BD literature poses considerable clinical implications.

Despite the literature, it remains unclear how alcohol use fluctuates over time in BD and how longitudinal dynamics interact with proximal changes in depressive, manic or hypomanic, and anxiety symptoms. Examining these dynamics can inform the mechanisms of how alcohol use plays a role in poorer outcomes in BD, when to intervene, and whether alcohol use affects mood symptoms even at subclinical levels. This understanding is critical given that (1) general clinical practices function on the perspective that alcohol is primarily used as self-medication with little longitudinal research to support this viewpoint and (2) extant research has largely focused on co-occurrence of AUD only.

The objective of the present study was to characterize the longitudinal patterns of alcohol use in one of the largest, ongoing cohort studies of BD, the Prechter Longitudinal Study of Bipolar Disorder (PLS-BD),^21,22^ and examine the temporal associations among alcohol use, mood, anxiety, and functioning over time. We hypothesized that (1) greater alcohol use is associated with more manic or hypomanic and depressive symptoms, (2) within-person increases in alcohol use are associated with an increase in manic or hypomanic and depressive symptoms at later time points, (3) within-person worsening of mood is associated with future increase in alcohol use, and (4) within-person increases in alcohol use are associated with worse functioning. Given the limited research on the association between alcohol use and anxiety in BD, we conducted exploratory analyses to examine these bidirectional associations.

## Methods

### Participants

Participants for this cohort study were drawn from the PLS-BD, which recruits via advertisements, psychiatric clinics, mental health centers, and community outreach events across Michigan.^21,22^ The University of Michigan Institutional Review Board (IRB) approved the PLS-BD. All participants provided written informed consent and receive an annual stipend for participation. There were no additional incentives offered for participation in this specific secondary data analysis. The IRB approval of PLS-BD applies to the present study, and all participants agreed to the use of their data in future secondary data analysis. We followed the Strengthening the Reporting of Observational Studies in Epidemiology (STROBE) reporting guideline.

The complete PLS-BD cohort currently consists of individuals enrolled for a median (IQR) of 9 (0-16) years. Enrollment, which started in February 2006, is rolling. The present study analyzed data collected from February 2006 to April 2022. Exclusion criteria were neurological disease and inability to interview without being intoxicated on alcohol or substances. Selected participants were those with a diagnosis of BDI or BDII who had been in the study for at least 5 years (eFigure 1 in Supplement 1). Healthy controls and those with other psychiatric diagnoses were not included in the present study. Self-reported race and ethnicity data were collected to enhance the generalizability of the PLS-BD findings to multiple identities. Diagnosis was assessed using the Diagnostic Interview for Genetic Studies.^23^ A team of at least 2 doctoral-level psychologists or psychiatrists (including S.H.S., M.G.M., I.F.T.) confirmed the diagnoses using *Diagnostic and Statistical Manual of Mental Disorders* (Fourth Edition, Text Revision) criteria and available medical and treatment history.

### Materials and Procedures

Comprehensive information on the study procedures for participants in the PLS-BD is available elsewhere.^21,22^ All self-reported measures for the PLS-BD were collected digitally using a secure, web-based research study platform (REDCap; Vanderbilt University).^24,25^ Materials and procedures for the current investigation are detailed herein.

Alcohol consumption, drinking behavior, and alcohol-related problems were measured with the Alcohol Use Disorders Identification Test (AUDIT)^26^ at baseline and every 6 months. The AUDIT score range is from 0 to 40, with 8 or higher indicating AUD is highly probable; 8 to 14 indicating hazardous or harmful drinking; and 15 to 40 indicating severe drinking or dependence. Internal consistency, calculated using Cronbach α, was excellent (α = .90). Participants completed 59% of delivered AUDIT assessments.

The Life Functioning Questionnaire (LFQ),^27^ developed to assess life functioning in individuals with BD, was administered at baseline and every 2 months. The LFQ measures functioning over the past month in 4 domains: leisure time with friends (LFQ friend); leisure time with family (LFQ family); duties at work, school, or activity center (LFQ work); and duties at home (LFQ home). Items in the questionnaire are rated using a Likert scale: 0 (does not spend a substantial amount of time in domain), 1 (no problems), 2 (mild problems), 3 (moderate problems), and 4 (severe problems). These scores are averaged across each of the 4 domains. Internal consistency for each LFQ subscale was good (α = .77-.84). Participants completed 57% of delivered LFQ assessments.

The presence and severity of self-reported depressive symptoms over the prior 2 weeks, manic or hypomanic symptoms over the prior week, and anxiety symptoms over the prior 2 weeks were measured using the 9-Item Patient Health Questionnaire (PHQ-9; score range: 0-27, with 5-9 indicating mild, 10-14 indicating moderate, 15-19 indicating moderately severe, and ≥20 indicating severe),^28^ the Altman Self-Rating Mania Scale (ASRM; score range: 0-20, with ≥6 indicating high probability of hypomania),^29^ and the 7-item Generalized Anxiety Disorder assessment scale (GAD-7; score range: 0-21, with 5-9 indicating mild, 10-14 indicating moderate, and 15-21 indicating severe).^30^ The internal consistencies of these instruments were excellent (α = .90) for PHQ-9, good (α = .82) for ASRM, and excellent (α = .92) for GAD-7. The PHQ-9, ASRM, and GAD-7 were administered at baseline and every 2 months, and their respective completion rates were 58%, 58%, and 41%.

### Statistical Analysis 

Summary statistics were estimated using the psych package in RStudio, V.2022.02.0+443 (RStudio).^31^ To model the temporal dynamics among alcohol use, mood, and functioning, we used Dynamic Structural Equation Modeling (DSEM)^32^ to fit multivariable, multilevel first-order vector autoregressive models in MPlus, version 8.1 (MPlus). A strength of DSEM is its ability to handle missingness in longitudinal data: DSEM uses the Kalman filter approach, which estimates a missing observation at time*_t_* based on its lagged observation.^33^ Details regarding DSEM are provided in eAppendix in Supplement 1. Seven unconditional models were fit for each measure (AUDIT with PHQ-9, ASRM, and GAD-7; 4 LFQ domains). These models yielded 8 parameters (2 means, autocorrelations, cross-lagged regressions, and variances) that were estimated simultaneously and allowed to vary by individual. For example, for the PHQ-9 and AUDIT model, each individual had the following 8 parameter scores: (1) μ_PHQ-9_, mean PHQ-9 over time; (2) μ*_AUDIT_*, mean AUDIT over time; (3) *ϕ_PP_*, mean association between PHQ-9 at time*_t_* and PHQ-9 6 months later *_t +1_*; (4) *ϕ_AA_*, mean association between AUDIT at time*_t_* and AUDIT 6 months later *_t +1_*; (5) *ϕ_AUDIT→_*_PHQ-9_, mean association between AUDIT at time*_t_* and PHQ-9 6 months later *_t +1_*; (6) ϕ_PHQ-9_*_→AUDIT_*, mean association between PHQ-9 at time*_t_* and AUDIT 6 months later*_t +1_*; (7) log(π_PHQ-9_), within-person variability in PHQ-9 over time; and (8) log(π*_AUDIT_*), within-person variability in AUDIT over time. All other models had the following parameters: *ϕ_MM_*, autocorrelation of ASRM; *ϕ_GG_*, autocorrelation of GAD-7; *ϕ_FF_*, autocorrelation of LFQ family (in LFQ family model); *ϕ_FF_*, autocorrelation of LFQ friend (in LFQ friend model); *ϕ_WW_*, autocorrelation of LFQ work; and *ϕ_HH_*, autocorrelation of LFQ home.

Conditional multivariable-multivariate models were then run to examine whether diagnostic status (0 = BDI, 1 = BDII), sex (0 = female, 1 = male), and time-varying covariates (age; use of mood stabilizers, antipsychotics, antidepressants, and benzodiazepines: 0 = no, 1 = yes) were associated with each of the 8 parameters. Results were evaluated based on the 95% credibility interval (CrI); CrIs that did not contain 0 were considered to be credible differences or associations.

## Results

This study included 584 participants (386 females (66.1%), 197 males (33.7%); mean [SD] age, 40 [13.6] years; 5 individuals self-reported as Asian [0.9%], 39 as Black or African American [6.7%], 21 as Hispanic or Latino [3.6%], 3 as Native American or Alaskan Native [0.5%], 511 as White [87.5%], and 15 as being of more than 1 race and ethnicity [2.6%], with 11 individuals not disclosing [1.9%]). Among these participants, 445 (76.2%) had a diagnosis of BDI and 139 (23.8%) had BDII (eFigure 1 in Supplement 1). All summary statistics (eTable 1 in Supplement 1), between-person and within-person zero-order correlations (eFigure 2 in Supplement 1), model fit indices (eTable 2 in Supplement 1), and unconditional model results (eTables 3-9 in Supplement 1) are provided in Supplement 1. Demographic statistics for the entire cohort are available elsewhere.^22^ Herein we report the results from the conditional models, including planned covariates.

### Longitudinal Patterns of Alcohol Use

**Figure 1.**
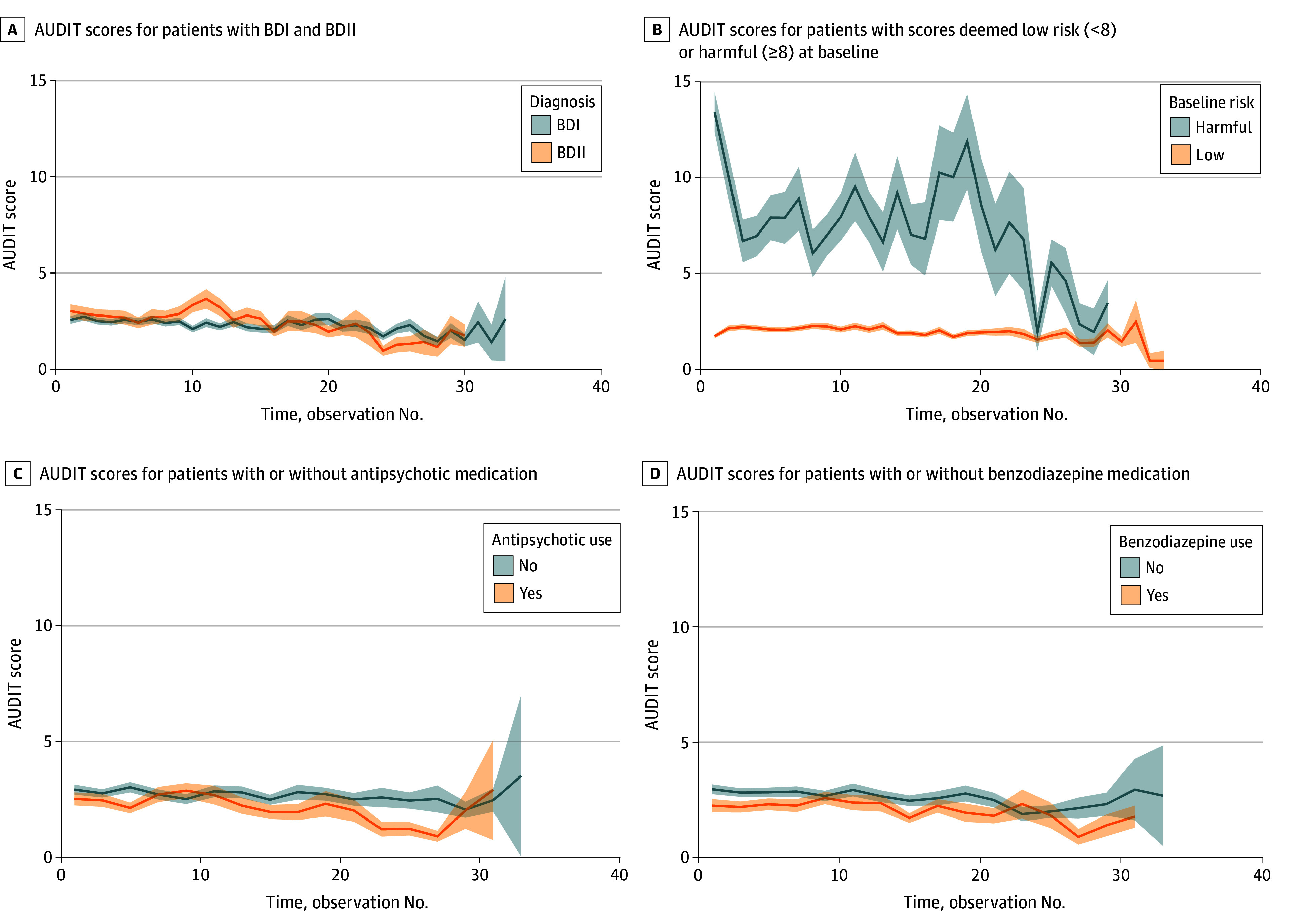
Longitudinal Patterns of Alcohol Use Disorder Shaded regions reflect the SE around the mean. AUDIT indicates Alcohol Use Disorders Identification Test; BDI, bipolar disorder type I; BDII, bipolar disorder type II. The AUDIT score range is from 0 to 40, with 8 or higher indicating AUD is highly probable; 8 to 14 indicating hazardous or harmful drinking; and 15 to 40 indicating severe drinking or dependence.

At baseline, 246 participants (42.1%) had a lifetime AUD. Across all models, alcohol use was highly variable over time; however, individuals differed in the intensity of this variability (Figure 1). The autocorrelation of alcohol use (ie, correlation of increase in problematic drinking at 1 time point with an increase at the next time point) was more pronounced in BDII than BDI (β = 0.10; 95% CrI, 0.03-0.19) and less pronounced for individuals taking benzodiazepines compared with individuals not taking benzodiazepines (β = –0.16; 95% CrI, –0.28 to –0.03) (Table 1). Lower variability in alcohol use was associated with older age (β = –0.14; 95% CrI, –0.19 to –0.07), antipsychotic use (β = –0.13; 95% CrI, –0.22 to –0.03), and benzodiazepine use (β = –0.14; 95% CrI, –0.23 to –0.05), whereas higher variability was associated with antidepressant use (β = 0.10; 95% CrI, 0.01-0.20). Antipsychotic use was associated with lower mean alcohol use (β = –0.18; 95% CrI, –0.30 to –0.08).

**Table 1.** Conditional Dynamic Structural Equation Modeling (DSEM) for Affective Measures^a^

Variables^b^	Estimated mean, β (95% CrI)	Between-person independent variables, estimated β (95% CrI)						
		Age	Sex	BD diagnosis	Mood stabilizer use	Antipsychotic use	Antidepressant use	Benzodiazepine use
Depression								
* μ_PHQ-9_*	8.15 (6.61 to 9.72)^c^	−0.09 (−0.16 to −0.02)^c^	−0.02 (−0.09 to 0.04)	0.08 (0.01 to 0.15)^c^	−0.16 (−0.26 to −0.04)^c^	0.03 (−0.09 to 0.12)	0.14 (0.04 to 0.25)^c^	0.13 (0.02 to 0.23)^c^
* μ_AUDIT_*	2.65 (1.98 to 3.33)^c^	−0.12 (−0.19 to −0.05)^c^	−0.02 (−0.09 to 0.05)	0.01 (−0.07 to 0.09)	0.04 (−0.07 to 0.15)	−0.18 (−0.30 to −0.08)^c^	0.06 (−0.06 to 0.16)	−0.11 (−0.21 to 0.00)
* ϕ_PP_*	0.29 (0.12 to 0.46)^c^	0.02 (−0.10 to 0.12)	0.08 (−0.04 to 0.20)	−0.09 (−0.20 to 0.03)	−0.21 (−0.38 to −0.04)^c^	0.10 (−0.08 to 0.25)	0.01 (−0.16 to 0.18)	0.03 (−0.14 to 0.18)
* ϕ_AA_*	0.51 (0.33 to 0.68)^c^	−0.13 (−0.22 to −0.05)^c^	−0.06 (−0.16 to 0.03)	0.10 (0.03 to 0.19)^c^	0.00 (−0.14 to 0.15)	0.03 (−0.10 to 0.17)	−0.03 (−0.17 to 0.10)	−0.16 (−0.28 to −0.03)^c^
* ϕ_AUDIT→PHQ-9_*	−0.07 (−0.27 to 0.15)	0.10 (−0.12 to 0.36)	−0.03 (−0.26 to 0.20)	0.12 (−0.13 to 0.43)	0.22 (−0.14 to 0.58)	0.06 (−0.29 to 0.54)	−0.32 (−0.62 to 0.01)	0.15 (−0.25 to 0.48)
* ϕ_PHQ-9→AUDIT_*	0.01 (−0.02 to 0.03)	−0.00 (−0.13 to 0.12)	0.01 (−0.12 to 0.12)	−0.11 (−0.24 to 0.02)	0.12 (−0.08 to 0.29)	−0.21 (−0.39 to −0.02)^c^	−0.00 (−0.19 to 0.19)	−0.01 (−0.17 to 0.17)
* log(π_PHQ-9_)*	2.76 (2.24 to 3.28)^c^	−0.05 (−0.13 to 0.02)	−0.07 (−0.14 to −0.00)^c^	0.06 (−0.01 to 0.13)	−0.16 (−0.28 to −0.05)^c^	0.04 (−0.06 to 0.15)	0.15 (0.03 to 0.25)^c^	0.12 (0.02 to 0.23)^c^
* log(π_AUDIT_)*	1.30 (0.29 to 2.29)^c^	−0.14 (−0.19 to −0.07)^c^	−0.02 (−0.08 to 0.04)	−0.01 (−0.07 to 0.06)	0.04 (−0.06 to 0.14)	−0.13 (−0.22 to −0.03)^c^	0.10 (0.01 to 0.20)^c^	−0.14 (−0.23 to −0.05)^c^
Mania								
* μ_ASRM_*	1.35 (0.66 to 2.04)^c^	0.06 (−0.01 to 0.13)	0.15 (0.08 to 0.23)^c^	−0.01 (−0.09 to 0.07)	0.00 (−0.12 to 0.11)	−0.05 (−0.15 to 0.07)	0.05 (−0.05 to 0.16)	0.19 (0.08 to 0.30)^c^
* ϕ_MM_*	0.21 (0.03 to 0.39)^c^	0.09 (−0.02 to 0.19)	0.01 (−0.10 to 0.12)	−0.10 (−0.19 to 0.01)	−0.03 (−0.19 to 0.13)	0.04 (−0.12 to 0.19)	−0.03 (−0.19 to 0.11)	−0.12 (−0.26 to 0.02)
* ϕ_AUDIT→ASRM_*	0.08 (−0.09 to 0.23)	−0.05 (−0.23 to 0.13)	−0.05 (−0.21 to 0.12)	0.16 (0.02 to 0.30)^c^	−0.12 (−0.38 to 0.12)	0.14 (−0.11 to 0.35)	−0.21 (−0.44 to 0.02)	0.11 (−0.12 to 0.32)
* ϕ_ASRM→AUDIT_*	−0.01 (−0.05 to 0.02)	0.08 (−0.08 to 0.22)	−0.06 (0.19 to −0.22)	−0.00 (−0.17 to 0.16)	0.15 (−0.08 to 0.38)	−0.06 (−0.30 to 0.18)	−0.02 (−0.25 to 0.21)	−0.11 (−0.31 to 0.09)
*log(π_ASRM_*)	1.20 (0.00.53 to 1.88)^c^	−0.00.01 (−0.07 to 0.06)	0.00 (−0.07 to 0.07)	0.01 (−0.05 to 0.07)	−0.05 (−0.16 to 0.06)	0.05 (−0.05 to 0.14)	−0.05 (−0.15 to 0.06)	0.14 (0.03 to 0.24)^c^
Anxiety								
* μ_GAD-7_*	8.03 (6.12 to 9.90)^c^	−0.16 (−0.23 to −0.08)^c^	−0.06 (−0.14 to 0.02)	0.08 (0.01 to 0.15)^c^	−0.14 (−0.25 to −0.03)^c^	0.04 (−0.08 to 0.15)	0.07 (−0.05 to 0.18)	0.29 (0.19 to 0.39)^c^
* ϕ_GG_*	0.19 (−0.08 to 0.44)	0.03 (−0.10 to 0.15)	0.06 (−0.08 to 0.19)	0.02 (−0.11 to 0.14)	−0.05 (−0.24 to 0.11)	0.04 (−0.13 to 0.20)	0.09 (−0.11 to 0.27)	−0.04 (−0.22 to 0.12)
* ϕ_AUDIT→GAD-7_*	0.19 (−0.02 to 0.46)	−0.15 (−0.40 to 0.11)	0.00 (−0.34 to 0.28)	−0.02 (−0.29 to 0.23)	−0.24 (−0.69 to 0.35)	0.07 (−0.35 to 0.53)	0.06 (−0.35 to 0.52)	−0.15 (−0.60 to 0.26)
* ϕ_GAD-7→AUDIT_*	−0.02 (−0.05 to 0.02)	0.11 (−0.06 to 0.26)	0.03 (−0.14 to 0.20)	−0.01 (−0.17 to 0.15)	0.06 (−0.19 to 0.27)	−0.13 (−0.38 to 0.11)	−0.03 (−0.26 to 0.21)	−0.03 (−0.23 to 0.17)
* log(π_GAD-7_)*	3.49 (2.74 to 4.19)^c^	−0.15 (−0.22 to −0.08)^c^	−0.08 (−0.16 to −0.01)^c^	−0.01 (−0.08 to 0.06)	−0.16 (−0.27 to −0.05)^c^	−0.07 (−0.18 to 0.05)	0.08 (−0.03 to 0.18)	0.17 (0.06 to 0.27)^c^

Abbreviations: ASRM, Altman Self-Rating Mania Scale; BD, bipolar disorder; CrI, credibility interval.

^a^ Estimated means ( 95% CrIs) represent the mean of that DSEM parameter across the sample. Estimates β (95% CrIs) include standardized estimates and 95% CrIs. For example, patients using anticonvulsant mood stabilizers had a mean depression score that was 0.16 (95% CrI, −0.26 to −0.04) SDs below the estimated mean (8.15; 95% CrI, 6.61-9.72). Note that *μ_AUDIT,_* ϕ_AA_, and log(π*_AUDIT_*) are not repeated in each model to reduce redundancy in the table.

^b^ See the Methods section for details on the parameters for the variables.

^c^ The 95% CrI does not include 0, suggesting that the association is credible.

### Affective Symptoms and Alcohol Use

**Figure 2.**
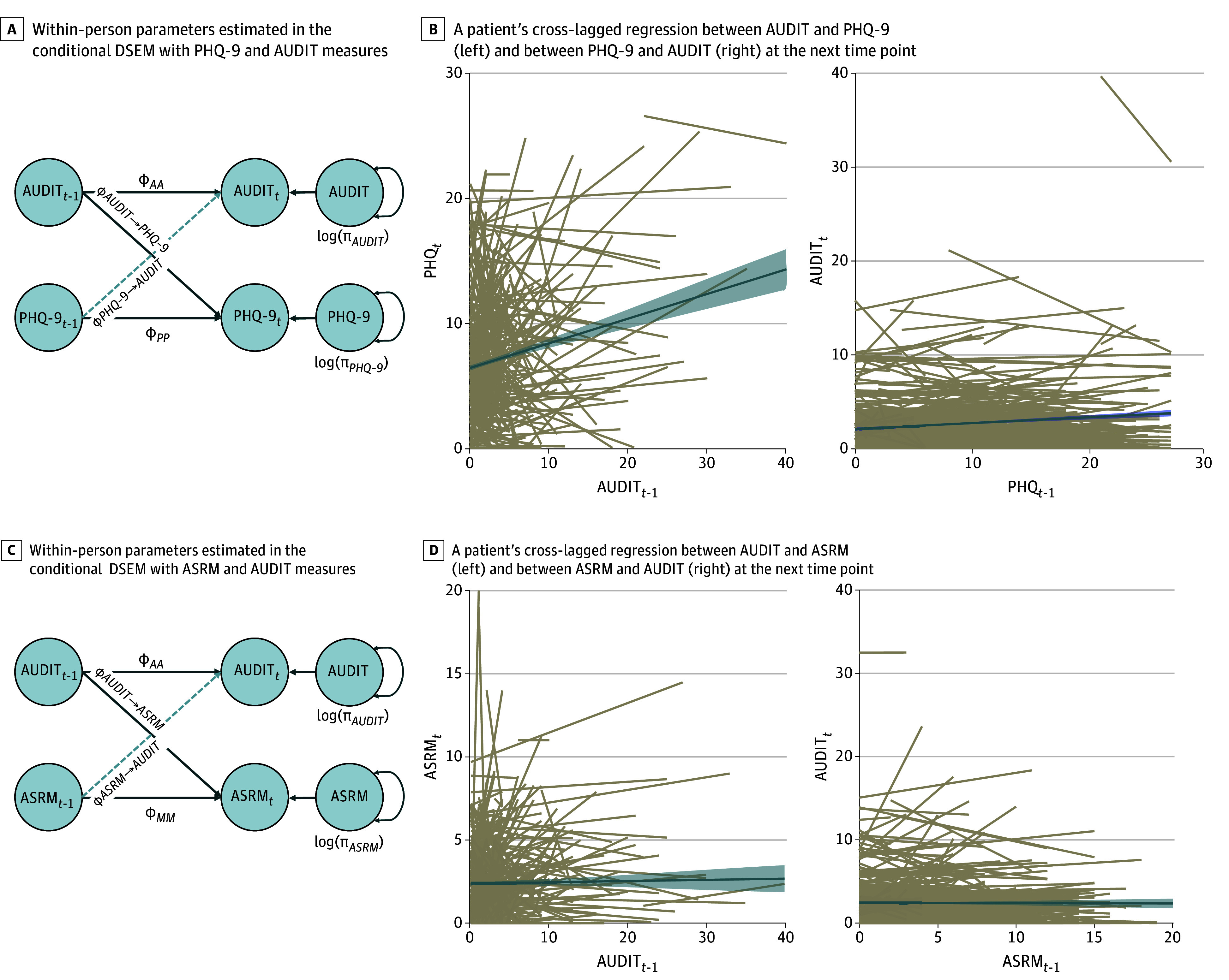
Conditional Dynamic Structural Equation Modeling (DSEM) for Affective Measures A and C, Dashed lines represent noncredible differences (95% CrI contains 0), and solid lines represent credible differences (95% CrI does not include 0). *t* indicates time; t-1, time – 1 observation; *ϕ_AA_*, autocorrelation of Alcohol Use Disorders Identification Test (AUDIT); *ϕ_ASRM→AUDIT_*, cross-lagged association between Altman Self-Rating Mania Scale (ASRM) and AUDIT at the next time point; *ϕ_AUDIT→_*_ASRM_, cross-lagged association between AUDIT and ASRM at the next time point; *ϕ_AUDIT→_*_PHQ-9_, cross-lagged association between AUDIT and 9-Item Patient Health Questionnaire (PHQ-9) at the next time point; *ϕ_MM_*, autocorrelation of ASRM; ϕ_PHQ-9_*_→AUDIT_*, cross-lagged association between PHQ-9 and AUDIT at the next time point; *ϕ_PP_*, autocorrelation of PHQ-9; log(π_ASRM_), within-person variability in ASRM; log(π*_AUDIT_*), within-person variability in AUDIT; log(π_PHQ-9_), within-person variability in PHQ-9. B and C, Each tan line represents a participant in the study. The blue solid line represents the group mean with SE around the mean.

A person reporting alcohol use above their own mean amount tended to experience more depressive symptoms at the next time point, but increased depressive symptoms were not associated with greater subsequent alcohol use (β = 0.04; 95% CrI, 0.01-0.07) (Figure 2). This cross-lagged association was not as pronounced for those taking antipsychotics compared with those not taking antipsychotics (β = –0.21; 95% CrI, –0.39 to –0.02) (Table 1). Previous depression symptoms were associated with 11.0% of the variance in alcohol use after 6 months, whereas alcohol use was associated with 34.0% of the variance in depression symptoms after 6 months. Regarding mania or hypomania, alcohol use exceeding one’s own mean amount was associated with an increase in manic or hypomanic symptoms at the next time point, but not vice versa (β = 0.04; 95% CrI, 0.01-0.07]) (Figure 2). Those with BDII showed a more pronounced cross-lagged association between alcohol use and manic or hypomanic symptoms compared with those with BDI (β = 0.16; 95% CrI, 0.02-0.30) (Table 1). Manic or hypomanic symptoms were associated with 11.0% of the variance in alcohol use after 6 months, whereas alcohol use was associated with 18.0% of the variance in manic or hypomanic symptoms after 6 months. Regarding anxiety, no within-person associations emerged with alcohol use, regardless of covariates (eFigure 3 in Supplement 1; Table 1).

### Functioning and Alcohol Use

**Figure 3.**
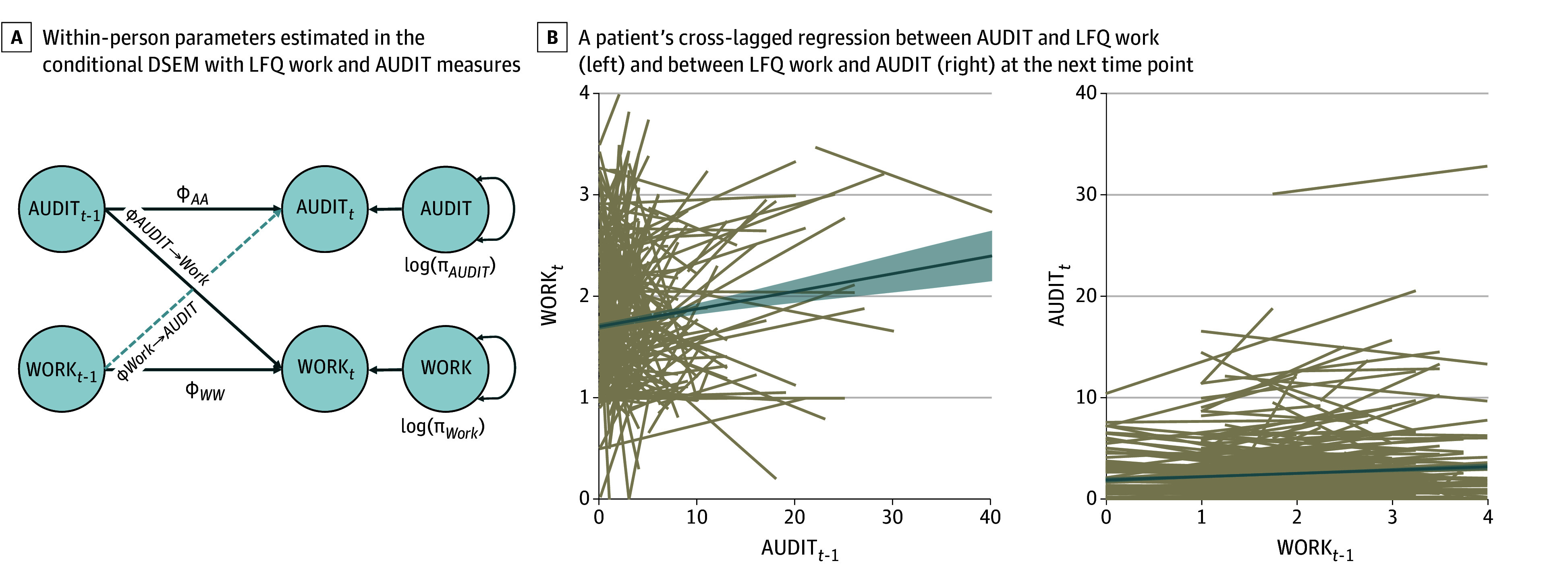
Conditional Dynamic Structural Equation Modeling (DSEM) for Life Functioning A, Dashed lines represent noncredible differences (95% credible interval [CrI] contains 0), and solid lines represent credible differences (95% CrI does not include 0). *t* indicates time; t-1, time – 1 observation; *ϕ_AA_*, autocorrelation of Alcohol Use Disorders Identification Test (AUDIT); *ϕ_AUDIT→Work_*, cross-lagged association between AUDIT and Life Functioning Questionnaire (LFQ) work at the next time point; ϕ_Work_*_→AUDIT_*, cross-lagged association between LFQ work and AUDIT at the next time point; log(π*_AUDIT_*), within-person variability in AUDIT; log(π*_Work_*), within-person variability of LFQ work; *ϕ_WW_*, autocorrelation of LFQ work. B, Each tan line represents a participant in the study. The blue solid line represents the group mean with SE around the mean.

A person reporting alcohol use above their own mean amount tended to experience a decrease in work functioning at the next time point, but not vice versa (β = 0.03; 95% CrI, 0.00-0.06) (Figure 3). This association was more pronounced in BDII than BDI (β = 0.26; 95% CrI, 0.06-0.45) and for individuals using mood stabilizers (β = 0.38; 95% CrI, 0.03-0.62), and it was less pronounced for individuals using antidepressants (β = −0.29; 95% CrI, −0.51 to −0.01) (Table 2). Given the large and near-0 95% CrI for the latter finding, caution should be exercised about overinterpreting the result. Work functioning was associated with 17.0% of the variance in alcohol use after 6 months, whereas alcohol use was associated with 40.0% of the variance in work functioning after 6 months. No within-person associations emerged between AUDIT scores and the family, friend, or home functioning domains (eFigure 4 in Supplement 1).

**Table 2.** Conditional Dynamic Structural Equation Modeling (DSEM) for Functioning Measures^a^

Variables^b^	Estimated mean, β (95% CrI)	Between-person independent variables, estimated β (95% CrI)						
		Age	Sex	BD diagnosis	Mood stabilizer use	Antipsychotic use	Antidepressant use	Benzodiazepine use
**Life Functioning Questionnaire family**								
*μ_Fam_*	1.55 (1.33 to 1.81)^c^	0.01 (−0.08 to 0.08)	−0.03 (−0.11 to 0.05)	−0.02 (−0.10 to 0.06)	−0.14 (−0.26 to −0.01)^c^	−0.01 (−0.13 to 0.11)	−0.06 (−0.18 to 0.07)	0.12 (−0.01 to 0.24)
*μ_AUDIT_*	2.32 (1.58 to 3.06)^c^	−0.09 (−0.16 to −0.02)^c^	−0.02 (−0.10 to 0.05)	0.01 (−0.06 to 0.09)	0.08 (−0.05 to 0.19)	−0.20 (−0.31 to −0.07)^c^	0.04 (−0.08 to 0.16)	−0.10 (−0.20 to 02)
*ϕ_FF_*	0.39 (0.17 to 0.59)^c^	−0.09 (−0.20 to 0.03)	0.05 (−0.07 to 0.16)	−0.09 (−0.20 to 0.02)	−0.13 (−0.30 to 0.05)	0.13 (−0.04 to 0.30)	0.01 (−0.17 to 0.18)	0.01 (−0.17 to 0.18)
*ϕ_AA_*	0.40 (0.21 to 0.61)^c^	−0.09 (−0.18 to 0.01)	−0.06 (−0.15 to 0.03)	0.14 (0.04 to 0.23)^c^	0.01 (−0.15 to 0.16)	0.03 (−0.12 to 0.18)	−0.05 (−0.20 to 0.09)	−0.21 (−0.34 to −0.06)^c^
*ϕ_AUDIT→Fam_*	0.01 (−0.04 to 0.06)	−0.10 (−0.33 to 0.11)	0.05 (−0.14 to 0.20)	0.04 (−0.18 to 0.26)	0.02 (−0.34 to 0.35)	0.23 (−0.07 to 0.49)	−0.15 (−0.43 to 0.14)	0.07 (−0.23 to 0.36)
*ϕ_Fam→AUDIT_*	−0.00 (−0.05 to 0.05)	−0.00 (−0.24 to 0.22)	0.03 (−0.20 to 0.28)	0.05 (−0.20 to 0.28)	0.02 (−0.31 to 0.33)	0.06 (−0.32 to 0.39)	−0.09 (−0.41 to 0.27)	0.02 (−0.30 to 0.33)
*log(π_Fam_)*	−1.37 (−2.02 to −0.71)^c^	0.04 (−0.04 to 0.10)	−0.05 (−0.13 to 0.01)	0.00 (−0.07 to 0.07)	−0.15 (−0.26 to −0.04)^c^	0.03 (−0.08 to 0.14)	0.04 (−0.07 to 0.15)	0.10 (−0.01 to 0.21)
*log(π_AUDIT_)*	0.61 (−0.47 to 1.75)	−0.15 (−0.21 to −0.09)^c^	−0.00 (−0.06 to 0.06)	0.02 (−0.04 to 0.08)	0.11 (0.00 to 0.21)^c^	−0.11 (−0.22 to −0.01)^c^	0.09 (−0.02 to 0.20)	−0.15 (−0.24 to −0.04)^c^
**Life Functioning Questionnaire friend**								
*μ_Fnd_*	1.25 (1.04 to 1.47)^c^	−0.06 (−0.14 to 0.02)	0.02 (−0.06 to 0.10)	0.05 (−0.03 to 0.13)	0.00 (−0.13 to 0.13)	0.08 (−0.05 to 0.21)	0.09 (−0.04 to 0.22)	0.02 (−0.12 to 0.14)
*ϕ_FF_*	0.34 (0.11 to 0.54)^c^	−0.10 (−0.20 to 0.02)	0.07 (−0.05 to 0.17)	−0.08 (−0.19 to 0.04)	−0.08 (−0.26 to 0.02)	0.19 (0.03 to 0.36)^c^	0.06 (−0.12 to 0.21)	0.07 (−0.1 to 0.24)
*ϕ_AUDIT→Fnd_*	0.01 (−0.03 to 0.05)	0.01 (−0.20 to 0.17)	−0.08 (−0.27 to 0.08)	0.04 (−0.15 to 0.22)	0.10 (−0.24 to 0.44)	−0.03 (−0.33 to 0.29)	−0.11 (−0.36 to 0.17)	−0.28 (−0.56 to 0.03)
*ϕ_Fnd→AUDIT_*	−0.00 (−0.05 to 0.05)	0.00 (−0.25 to 0.23)	−0.03 (−0.26 to 0.22)	0.01 (−0.24 to 0.24)	0.03 (−0.29 to 0.35)	−0.02 (−0.41 to 0.32)	0.06 (−0.28 to 0.42)	−0.02 (−0.34 to 0.29)
*log(π_Fnd_)*	−1.68 (−2.35 to −1.05)^c^	0.01 (−0.06 to 0.07)	−0.03 (−0.10 to 0.04)	−0.00 (−0.07 to 0.06)	−0.14 (−0.24 to −0.03)^c^	0.14 (0.03 to 0.24)^c^	0.09 (−0.02 to 0.19)	0.11 (0.01 to 0.23)^c^
**Life Functioning Questionnaire work**								
*μ_Work_*	1.63 (1.38 to 1.90)^c^	0.03 (−0.05 to 0.10)	−0.08 (−0.15 to −0.01)^c^	0.02 (−0.06 to 0.09)	−0.16 (−0.27 to −0.04)^c^	0.06 (−0.05 to 0.18)	0.23 (0.12 to 0.34)^c^	0.08 (−0.04 to 0.19)
*ϕ_WW_*	0.33 (0.13 to 0.54)^c^	−0.09 (−0.20 to 0.04)	0.14 (0.01 to 0.25)^c^	−0.04 (−0.16 to 0.08)	−0.09 (−0.27 to 0.10)	0.03 (−0.15 to 0.19)	0.11 (−0.07 to 0.29)	−0.15 (−0.33 to 0.02)
*ϕ_AUDIT→Work_*	−0.04 (−0.07 to −0.00)^c^	0.11 (−0.11 to 0.29)	0.03 (−0.14 to 0.21)	0.26 (0.06 to 0.45)^c^	0.38 (0.03 to 0.62)^c^	0.05 (−0.27 to 0.36)	−0.29 (−0.51 to −0.01)^c^	−0.03 (−0.31 to 0.27)
*ϕ_Work→AUDIT_*	−0.00 (−0.05 to 0.05)	−0.00 (−0.24 to 0.23)	0.04 (−0.21 to 0.28)	0.03 (−0.21 to 0.27)	0.10 (−0.24 to 0.40)	−0.11 (−0.46 to 0.22)	−0.03 (−0.35 to 0.32)	0.04 (−0.28 to 0.34)
*log(π_Work_)*	−1.85 (−2.41 to −1.29)^c^	0.08 (0.01 to 0.15)^c^	−0.14 (−0.21 to −0.07)^c^	−0.00 (−0.07 to 0.06)	−0.08 (−0.19 to 0.04)	0.07 (−0.03 to 0.19)	0.16 (0.05 to 0.26)^c^	0.01 (−0.10 to 0.13)
**Life Functioning Questionnaire home**								
*μ_Home_*	1.90 (1.67 to 2.17)^c^	−0.35 (−0.42 to −0.28)^c^	−0.04 (−0.10 to 0.03)	0.02 (−0.05 to 0.08)	−0.04 (−0.17 to 0.06)	−0.05 (−0.16 to 0.05)	0.06 (−0.05 to 0.17)	−0.21 (−0.32 to −0.11)^c^
*ϕ_HH_*	0.23 (0.01 to 0.43)^c^	0.02 (−0.10 to 0.15)	0.04 (−0.08 to 0.15)	−0.03 (−0.14 to 0.08)	−0.13 (−0.31 to 0.05)	0.12 (−0.05 to 0.28)	0.05 (−0.12 to 0.21)	−0.07 (−0.24 to 0.12)
*ϕ_AUDIT→Home_*	0.00 (−0.03 to 0.04)	−0.13 (−0.31 to 0.08)	0.04 (−0.14 to 0.21)	0.06 (−0.17 to 0.27)	0.07 (−0.22 to 0.33)	0.09 (−0.17 to 0.34)	0.09 (−0.18 to 0.35)	0.03 (−0.26 to 0.31)
*ϕ_Home→AUDIT_*	0.00 (−0.06 to 0.06)	−0.05 (−0.29 to 0.19)	−0.02 (−0.29 to 0.24)	0.01 (−0.29 to 0.31)	0.05 (−0.37 to 0.41)	0.01 (−0.43 to 0.39)	0.08 (−0.29 to 0.44)	−0.04 (−0.41 to 0.33)
*log(π_Home_)*	0.05 (−0.73 to 0.87)	−0.16 (−0.23 to −0.10)^c^	−0.09 (−0.15 to −0.02)^c^	−0.05 (−0.11 to 0.01)	−0.03 (0.13 to 0.08)	0.00 (−0.10 to 0.11)	0.10 (−0.01 to 0.20)	−0.15 (−0.26 to −0.05)^c^

Abbreviations: BD, bipolar disorder; CrI, credibility interval.

^a^ Estimated means (95% CrIs) represent the mean of that DSEM parameter across the sample. Estimated β (95% CrIs) include standardized estimates and 95% CrIs. For example, patients using antipsychotic mood stabilizers had a mean Alcohol Use Disorders Identification Test score that was −0.20 (95% CrI, −0.31 to −0.07) SD below the estimated mean (2.32; 95% CrI, 1.58-3.06). Note that *μ_AUDIT_*, ϕ_AA_, and log(π*_AUDIT_*) are not repeated in each model to reduce redundancy in the table.

^b^ See the Methods section for details on the parameters for the variables.

^c^ The 95% CrI does not include 0, suggesting that the association is credible.

## Discussion

Consistent with our hypotheses, more problematic drinking was associated with depression and mania or hypomania later. Increased depression and mania or hypomania was not associated with greater subsequent alcohol use. Furthermore, greater problematic alcohol use was associated with worse functioning at work but was not associated with functioning in other domains later. Taken together, these results highlight the role that alcohol use may play in ongoing mood instability and functional impairment in BD.

The findings regarding the temporal association of depression and mania or hypomania with alcohol use are inconsistent with the self-medication hypothesis of addiction.^34^ The self-medication hypothesis posits that addiction is associated with use of substances to mitigate distressing symptoms. Thus, in individuals with psychiatric disorders, the behavioral motivator of substance use may be to reduce or increase specific emotional states.^35^ Alcohol, given its fast action and temporary relief as a central nervous system depressant, may be particularly prone to such use. By this logic, we would have expected that increased affective symptoms would have been associated with greater alcohol use over time. However, we found that the opposite (ie, increased problematic alcohol use) was associated with subsequent worsening of depressive and manic or hypomanic symptoms. Notably, there were no significant individual differences in this random effect, indicating that this temporal pattern was consistent across individuals.

We also found diagnostic differences in alcohol use. Individuals with BDII exhibited higher autocorrelation in AUDIT scores, indicating that greater alcohol use in this group was more likely to persist over time. These individuals also exhibited more pronounced worsening of workplace function following a period of increased problematic alcohol use. Increased manic or hypomanic symptoms after increased alcohol use were more pronounced in BDII than in BDI. Prior literature has linked greater alcohol use to mania or hypomania in BD in combined samples,^12,36,37^ but these studies did not examine whether these associations differed across subtypes. By conducting this delineated analysis, we provide insights into subtype-specific dynamics of alcohol use and mania or hypomania. However, the diagnostic findings seemingly contradict earlier studies that linked co-occurring alcohol use with BDI to a worse illness course than with BDII.^9,12^ While these previous studies assessed illness course in different ways, the present study of autocorrelation of alcohol use and functioning focused on previously unexplored domains.

Medication use emerged as a factor associated with the temporal dynamics of alcohol use. Individuals using antipsychotics reported lower mean AUDIT scores, less variable alcohol use, and a lower likelihood of depression playing a role in future alcohol use. Individuals using mood stabilizers exhibited more pronounced worsening of work functioning after a period of greater problematic alcohol use. Benzodiazepine use was associated with a lower autocorrelation in alcohol use but higher variability in depression and more manic or hypomanic symptoms. In contrast, individuals using antidepressants tended to have greater variability in alcohol use. Notably, these results should not be interpreted as causal as they were simply associations. It is plausible that persons with co-occurring BD and alcohol use are more likely to be prescribed antipsychotics or that individuals with higher depression and mania or hypomania are more likely to be using benzodiazepines during periods of higher symptom severity. Nonetheless, these medication-related findings emphasize the need for careful consideration of medication regimens in managing patients with BD who drink alcohol.

We did not find within-person or between-person associations between alcohol use and anxiety. Because this study used a longer timescale, the length of time between each assessment period may have concealed the temporal associations between a change in anxiety and change in drinking. For example, alcohol may be used as an acute remedy to anxiety in specific instances, thereby reducing the likelihood that it would emerge in a model using 6-month increments. Studies that use in-the-moment methods, such as ecological momentary assessment, might be better suited for examining the more proximal bidirectional associations between alcohol use and anxiety.^38^

While ecological momentary assessment could provide more proximal insights, the present study’s 6-month timescale has strengths. First, it enabled us to examine long-term patterns of alcohol use (as opposed to individual alcohol binge episodes) and mood (which is more prolonged than emotion). Furthermore, it allowed us to examine the implications of age for temporal dynamics of alcohol use in BD, which, on average, becomes less problematic and variable. Nonetheless, administering the AUDIT more frequently may help dissect the temporal association between increased alcohol use and mood. Although we found that increased alcohol use was associated with later mood symptoms, it remains plausible that this alcohol use was preceded by the beginning stages of a mood episode that the measures did not capture.

These findings hold critical clinical implications. Fluctuations in alcohol use were common in the sample and were associated with unstable mood and functional impairment at work, regardless of whether an individual had AUD. Thus, regular monitoring of alcohol use with the AUDIT or similar tools may be advisable in treating patients with BD. Relatedly, patients with BD may benefit from reduced alcohol consumption if their treatment goals included improving work functioning and/or reducing mood symptoms, complemented by other skills to prevent future problematic alcohol use. Notably, many individuals without AUD reported AUDIT scores that may benefit from intervention (eg, score of 8-14); thus, interventions focused on alcohol use in BD may be considered for anyone engaging in problematic alcohol use regardless of diagnostic status.

Because little research has evaluated integrated treatments for alcohol use and BD,^6,39^ it is unclear what kind of alcohol use treatment would be most helpful in BD. Future studies are needed to examine whether abstinence (refraining from any alcohol use) vs harm-reduction methods (self-moderation and reducing frequency or amount)^40^ differentially alter mood, functioning, and course of illness. Follow-up studies could focus on identifying motives or risk factors that may precede increases in alcohol use. Just-in-time adaptive interventions in combination with passive sensing technology could be deployed to a person who engages in alcohol risk behaviors. In sum, the present findings provide multiple avenues for future clinical intervention and research.

### Limitations

This study was limited by several aspects of the PLS-BD protocol and overall participant demographic makeup. First, this study was naturalistic in design, and therefore it is unclear whether specific types and timing of treatment (eg, medication changes and therapy) changed the longitudinal dynamics of alcohol use, mood, and functioning. Second, the PLS-BD cohort lacks racial diversity; most participants are White individuals. Although this demographic makeup mirrors that of the PLS-BD recruitment catchment area, it does not reflect the demographic characteristics of the US as a whole and therefore may limit the findings’ generalizability.

## Conclusions

The findings of this study suggest that alcohol use, regardless of AUD diagnostic status, may destabilize the course of BD, as it was associated with mood instability and poorer work functioning and did not appear to be a response to mood symptom changes. Baseline measures of the co-occurrence between BD and AUD diagnoses were not sufficient to capture the dynamic nature of alcohol use in this patient population. Greater efforts toward the dimensional and longitudinal assessment and management of alcohol use are necessary and should be integrated into research and standard treatment for individuals with BD.

## Notes

Supplement 1. **eFigure 1.** Subsample Selection and Sample Size **eAppendix.** DSEM Modeling Approach **eTable 1.** Demographic and Summary Statistics **eFigure 2.** Within and Between Person Spearman Rho Correlations **eTable 2.** Potential Scale Reduction and Deviance Information Criterion **eTable 3.** Unconditional DSEM Model: PHQ9 and AUDIT **eTable 4.** Unconditional DSEM Model: ASRM and AUDIT **eTable 5.** Unconditional DSEM Model: GAD7 and AUDIT **eTable 6.** Unconditional DSEM Model: LFQ Family and AUDIT **eTable 7.** Unconditional DSEM Model: LFQ Friend and AUDIT **eTable 8.** Unconditional DSEM Model: LFQ Family and AUDIT **eTable 9.** Unconditional DSEM Model: LFQ Work and AUDIT **eFigure 3.** Conditional DSEM Model for GAD7 and AUDIT **eFigure 4.** Conditional DSEM Model for LFQ Family, Friend, and Home with AUDIT

Supplement 2.
Data Sharing Statement
