# Supplementary material for: Longitudinal Interplay Between Alcohol Use, Mood, and Functioning in Bipolar Spectrum Disorders
Source: JAMA Netw Open. 2024 Jun 7;7(6):e2415295. doi: 10.1001/jamanetworkopen.2024.15295 (PMC11161848; doi:10.1001/jamanetworkopen.2024.15295)
Supplement: Supplement 1. — eFigure 1. Subsample Selection and Sample Size eAppendix. DSEM Modeling Approach eTable 1. Demographic and Summary Statistics eFigure 2. Within and Between Person Spearman Rho Correlations eTable 2. Potential Scale Reduction and Deviance Information Criterion eTable 3. Unconditional DSEM Model: PHQ9 and AUDIT eTable 4. Unconditional DSEM Model: ASRM and AUDIT eTable 5. Unconditional DSEM Model: GAD7 and AUDIT eTable 6. Unconditional DSEM Model: LFQ Family and AUDIT eTable 7. Unconditional DSEM Model: LFQ Friend and AUDIT eTable 8. Unconditional DSEM Model: LFQ Family and AUDIT eTable 9. Unconditional DSEM Model: LFQ Work and AUDIT eFigure 3. Conditional DSEM Model for GAD7 and AUDIT eFigure 4. Conditional DSEM Model for LFQ Family, Friend, and Home with AUDIT [file jamanetwopen-e2415295-s001.pdf]

Supplemental Online Content

Sperry SH, Stromberg AR, Murphy VA, et al. Longitudinal interplay between alcohol use, mood, and functioning in bipolar spectrum disorders. *JAMA Netw Open*. 2024;7(6):e2415295. doi:10.1001/jamanetworkopen.2024.15295

eFigure 1. Subsample Selection and Sample Size
eAppendix. DSEM Modeling Approach
eTable 1. Demographic and Summary Statistics
eFigure 2. Within and Between Person Spearman Rho Correlations
eTable 2. Potential Scale Reduction and Deviance Information Criterion
eTable 3. Unconditional DSEM Model: PHQ9 and AUDIT
eTable 4. Unconditional DSEM Model: ASRM and AUDIT
eTable 5. Unconditional DSEM Model: GAD7 and AUDIT
eTable 6. Unconditional DSEM Model: LFQ Family and AUDIT
eTable 7. Unconditional DSEM Model: LFQ Friend and AUDIT
eTable 8. Unconditional DSEM Model: LFQ Family and AUDIT
eTable 9. Unconditional DSEM Model: LFQ Work and AUDIT
eFigure 3. Conditional DSEM Model for GAD7 and AUDIT
eFigure 4. Conditional DSEM Model for LFQ Family, Friend, and Home with AUDIT

This supplemental material has been provided by the authors to give readers additional information about their work.

© 2024 Sperry SH et al. *JAMA Network Open*

eFigure1. Subsample selection and sample size

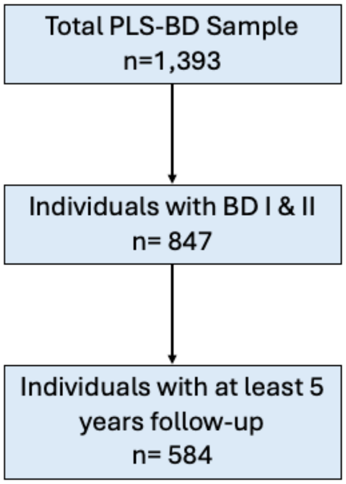

Note. PLS-BD = Prechter Longitudinal Study of Bipolar Disorder. Sample numbers as of April 2022 at data extraction.

eAppendix

DSEM Modeling Approach

DSEM decomposes within- and between-person variance using latent mean centering and allows for multiple random effects and their covariances to be modeled simultaneously. These Bayesian models use Markov chain Monte Carlo (MCMC) to estimate posterior distributions. MPlus' default settings adaptively assess convergence by drawing a minimum of 1,000 samples over two chains (with thinning = 10 and the first half of samples discarded as "burn-in") and iteratively assessing "Potential Scale Reduction" values (PSR)[32] for all parameters until they reach an acceptable range (i.e., <1.1). Following recommended procedures,[5] we re-ran each model with a larger number of samples[33] (50,000 iterations over 2 chains with thinning = 10, resulting in 5,000 MCMC samples [first 2,500 discarded as "burn-in"]); we evaluated models using standard diagnostics (trace/autocorrelation plots and PSR values); and, when diagnostics indicated poor parameter stability, we used a best-practice model building approach to decide upon final models that would produce reliable estimates.[34]

First, we estimated unconditional models. These models fit eight parameters for each individual including means, autocorrelations, cross-lagged regressions, and variability for each of the two variables defined in the model. Means represent the individuals average in each variable over their entire time series. Autocorrelations (inertia) reflects the extent to which a variable is associated with its score at the subsequent time point. Conceptually, a high autocorrelation could mean two things: (1) that when an individual has a deviation from their own average, they are more likely to retain that deviation at the next time point, or (2) their score at one point in time is highly correlated with their score at the next time point (regardless of whether the score itself is high or low). Cross-lagged relationships are of primary interest in these models. They reflect the extent to which a change in one variable at time t is associated with a change in another variable at time t+1. For example, a significant positive cross-lagged association between AUDIT and PHQ9 at the next time point means that when an individual has a higher AUDIT score than is typical for them (across the entire time-series), they are more likely to show a higher depression score than typical for them at the next time point. One of the benefits of the DSEM model is that we can assess bidirectionality of relationships. While these methods are correlative in nature, should one cross-lagged regression be significant in one direction but not the other, it can begin to speak to directionality of associations.

Next, we estimated multivariable – multivariate conditional models. In these models, we add independent variables (e.g., age, sex, diagnosis, medication status) with the eight parameters identified in the unconditional model as outcomes. For example, we can ask, does the cross-lagged regression between AUDIT and later PHQ9 look different for men vs. women, for those with BD I vs. BD II, or for those on a certain medication. In this respect, this process can be thought of traditional multilevel modeling or structural equation modeling and adding covariates or moderators to paths specified. Note that in the pre-registration we included those with BD Not Otherwise Specified and Schizoaffective Bipolar Subtype. However, the sample size of these diagnostic categories was too small to calculate group differences in parameters. As a result, we ran all models on only those with BD I and II. An example diagram showing a model for the PHQ9 (Dep) and AUDIT (Aud) is shown below.

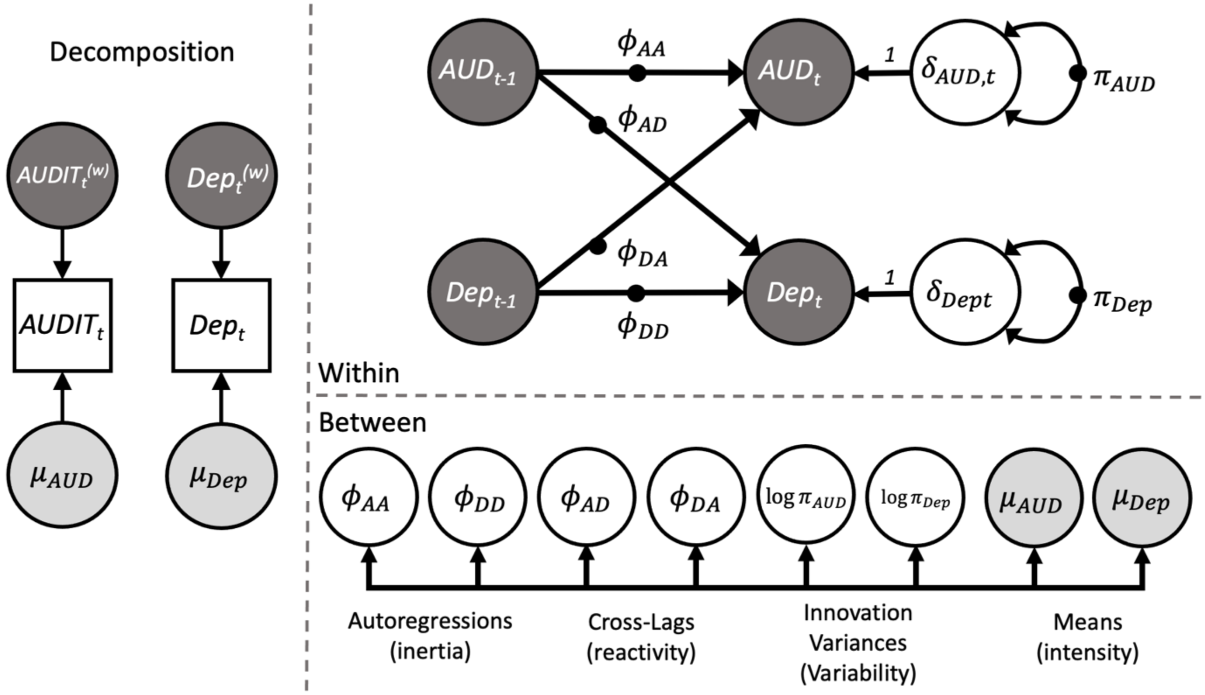

Example DSEM: (w) = within-individual. Example path diagram for unconditional bivariate multilevel VAR(1) model. Eight random effects will be specified: two individual differences each (AUD = AUDIT, Dep = PHQ9) for mean intensity, autoregressions (inertia), cross-lagged regressions (bidirectional relationships), innovation variances (variability).

© 2024 Sperry SH et al. *JAMA Network Open*

e**Table 1.**

Demographic and Summary Statistics

Variable	Total (N = 584)		
**Diagnosis**			
BDI	445 (76.2%)		
BDII	139 (23.8%)		
**Current Age**			
Mean (SD)	51.7 (13.9)		
Median [Min, Max]	50.0 [24.0, 93.0]		
**Age at Entrance to Cohort**			
Mean (SD)	40.0 (13.6)		
Median [Min, Max]	38.0 [0, 84.0]		
**Gender**			
Female	386 (66.1%)		
Male	197 (33.7%)		
Other	1 (0.2%)		
**Race**			
African-American	39 (6.7%)		
Asian	5 (0.9%)		
More Than One Race	15 (2.6%)		
Native American/Alaskan Native	3 (0.5%)		
Unknown or not reported	11 (1.9%)		
White or Caucasian	511 (87.5%)		
**Ethinicity**			
Hispanic or Latino	21 (3.6%)		
Not Hispanic or Latino	554 (94.9%)		
Unknown (Individuals not reporting ethnicity	9 (1.5%)		
	Median	**Range**	**Interquartile Range (IQR)**
**Depression**			
PHQ9	6	0 – 27	9
HAMD	6	0 – 31	10
**Mania**			
ASRM	1	0 – 14.5	4
YMRS	1	0 – 23	4

© 2024 Sperry SH et al. *JAMA Network Open*

**Anxiety**			
GAD7	6	0 – 21	9
**Alcohol Impairment**			
AUDIT	1	0 – 32.22	3
**Life Functioning**			
Friends	1	0 – 3.5	0.67
Family	1	0 – 4	1
Work	1.75	0 – 4	1.5
Home	1	0 – 3.5	1.5

Note. Mean scores for depression, mania, anxiety, AUDIT, and life functioning represent averages across longitudinal follow-up.

© 2024 Sperry SH et al. *JAMA Network Open*

eFigure 2. Within and Between Person Spearman Rho Correlations

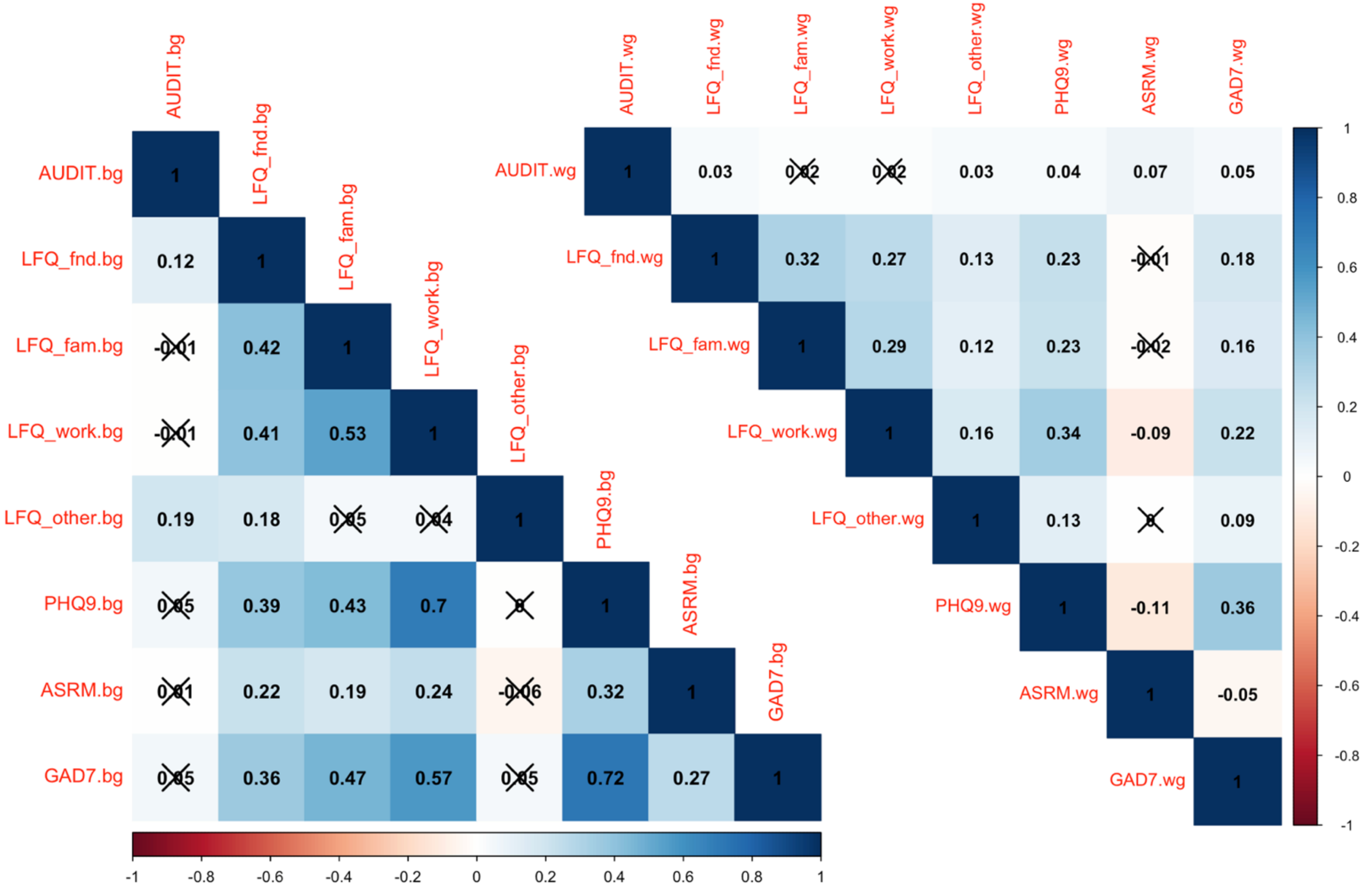

Note. The suffix .bg represents between-person associations whereas .wg represents within-person associations. Coefficients with an X represent those that were not significant (p<.05). Within- and between- person correlations were estimated using the Statsby package in R. Correlation coefficients are Spearman rho coefficients to account for variables that have positive or negative skew. Boxes that are blue correspond to positive correlations and boxes that are red correspond to negative correlations. The darker/more opaque a box is, the stronger the association.

© 2024 Sperry SH et al. *JAMA Network Open*

eTable 2. Potential Scale Reduction and Deviance Information Criterion

	Final Potential Scale Reduction (PSR)	Deviance Information Criteria (DIC)
Unconditional Models		
PHQ9 & AUDIT	1.03	75367.14
ASRM & AUDIT	1.08	54196.78
GAD7 & AUDIT	1.03	74238.12
LFQ Family & AUDIT	1.05	28582.29
LFQ Friend & AUDIT	1.04	24548.66
LFQ Home & AUDIT	1.06	23649.10
LFQ Work & AUDIT	1.05	29220.63
Conditional Models w/ covariates		
PHQ9 & AUDIT	1.09	141614.41
ASRM & AUDIT	1.07	128420.89
GAD7 & AUDIT	1.06	139087.12
LFQ Family & AUDIT	1.01	64215.02
LFQ Friend & AUDIT	1.02	62617.80
LFQ Home & AUDIT	1.05	60307.11
LFQ Work & AUDIT	1.01	64767.21

Note. Potential Scale Reduction (PSR) values are provided in Markov chain Monte Carlo and Bayesian models to assess convergence between the two chains being estimated. If the MCMC chains converge, PSR values should be < 1.1. Deviance Information Criteria (DIC) is a goodness of fit index provided in Mplus for DSEM modeling. Lower deviance indicates better fit.

© 2024 Sperry SH et al. *JAMA Network Open*

eTable 3. Unconditional DSEM model: PHQ9 and AUDIT

	Unstandardized Point Estimates		Standardized Estimates	
**Variable**	**Mean [SD]**	**95% Credibility Interval**	**Estimate [Posterior S.D]**	**95% Credibility Interval**
μ_{PHQ-9}	**7.38 [3.96]**	**6.99 – 7.80**	--	--
μ_{AUDIT}	**1.93 [1.55]**	**1.77 – 2.09**	--	--
ϕ_{PP}	**.24 [.28]**	**.19 – .28**	**.24 [.03]**	**.19 – .27**
ϕ_{AA}	**.33 [.28]**	**.29 – .36**	**.33 [.02]**	**.29 – .35**
$\phi_{AUDIT \to PHQ-9}$	.08 [.19]	-.02 – .21	**.04 [.01]**	**.02 – .07**
$\phi_{PHQ-9 \to AUDIT}$	-.01 [.03]	-.01 – .00	-.01 [.02]	-.04 – .02
$\log(\pi_{PHQ-9})$	**2.67 [1.15]**	**2.57 – 2.78**	**.85 [.03]**	**.81 – .87**
$\log(\pi_{AUDIT})$	**-.55 [2.86]**	**-.80 – -.30**	**.76 [.01]**	**.74 – .76**

Note. Unstandardized Point Estimates from Unconditional Models reflects the mean, standard deviation, and 95% credibility interval of the eight model parameters estimated in the unconditional model. Standardized estimates reflect the within-person associations of each of the eight parameters. 95% credibility intervals that do not contain zero are viewed as credible differences. PP= PHQ9 autocorrelation, AA = AUDIT autocorrelation, $\phi_{PHQ-9 \to AUDIT}$ represents the cross-lagged regression, and $\log(\pi_{PHQ-9})$ represents within-person variability.

© 2024 Sperry SH et al. *JAMA Network Open*

eTable 4. Unconditional DSEM model: ASRM and AUDIT

	Unstandardized Point Estimates		Standardized Estimates	
**Variable**	**Mean [SD]**	**95% Credibility Interval**	**Estimate [Posterior S.D]**	**95% Credibility Interval**
μ_{ASRM}	**2.41 [1.63]**	**2.24 – 2.59**	--	--
μ_{AUDIT}	**1.90 [1.46]**	**1.74 – 2.07**	--	--
ϕ_{MM}	**.24 [.29]**	**.18 – .28**	**.23 [.02]**	**.19 – .27**
ϕ_{AA}	**.33 [.28]**	**.29 – .36**	**.32 [.02]**	**.30 – .35**
$\phi_{AUDIT \to ASRM}$	**.13 [.14]**	**.06 – .19**	**.04 [.01]**	**.02 – .07**
$\phi_{ASRM \to AUDIT}$	.01 [.03]	-.00 – .03	.02 [.02]	-.01 – .05
$\log(\pi_{ASRM})$	**1.27 [1.66]**	**1.10 – 1.42**	**.83 [.02]**	**.80 – .86**
$\log(\pi_{AUDIT})$	**-.54 [2.88]**	**-.79 – -.29**	**.79 [.01]**	**.76 – .81**

Note. Unstandardized Point Estimates from Unconditional Models reflects the mean, standard deviation, and 95% credibility interval of the eight model parameters estimated in the unconditional model. Standardized estimates reflect the within-person associations of each of the eight parameters. 95% credibility intervals that do not contain zero are viewed as credible differences. MM = ASRM autocorrelation, AA = AUDIT autocorrelation, $\phi_{ASRM \to AUDIT}$ represents the cross-lagged regression, and $\log(\pi_{ASRM})$ represents within-person variability.

© 2024 Sperry SH et al. *JAMA Network Open*

eTable 5. Unconditional DSEM model: GAD7 and AUDIT

	Unstandardized Point Estimates		Standardized Estimates	
**Variable**	**Mean [SD]**	**95% Credibility Interval**	**Estimate [Posterior S.D]**	**95% Credibility Interval**
μ_{GAD7}	**6.57 [3.92]**	**6.10 – 7.02**	--	--
μ_{AUDIT}	**1.92 [1.53]**	**1.76 – 2.09**	--	--
ϕ_{GG}	**.32 [.32]**	**.29 – .36**	**.32 [.04]**	**.22 – .38**
ϕ_{AA}	**.33 [.28]**	**.29 – .36**	**.33 [.02]**	**.29 – .35**
$\phi_{AUDIT \rightarrow GAD7}$	-.12 [.22]	-.24 – .07	.00 [.01]	-.04 – .05
$\phi_{GAD7 \rightarrow AUDIT}$	**-.02 [.03]**	**-.04 – -.01**	**-.07 [.02]**	**-.11 – -.03**
$\log(\pi_{GAD7})$	**2.08 [1.59]**	**1.10 – 1.42**	**.76 [.04]**	**.69 – .80**
$\log(\pi_{AUDIT})$	**-.56 [2.86]**	**-.82 – -.29**	**.76 [.02]**	**.73 – .78**

Note. Unstandardized Point Estimates from Unconditional Models reflects the mean, standard deviation, and 95% credibility interval of the eight model parameters estimated in the unconditional model. Standardized estimates reflect the within-person associations of each of the eight parameters. 95% credibility intervals that do not contain zero are viewed as credible differences. GG = GAD7 autocorrelation, AA = AUDIT autocorrelation, $\phi_{GAD7 \rightarrow AUDIT}$ represents the cross-lagged regression, and $\log(\pi_{GAD7})$ represents within-person variability.

© 2024 Sperry SH et al. *JAMA Network Open*

eTable 6. Unconditional DSEM model: LFQ Family and AUDIT

	Unstandardized Point Estimates		Standardized Estimates	
**Variable**	**Mean [SD]**	**95% Credibility Interval**	**Estimate [Posterior S.D]**	**95% Credibility Interval**
μ_{Fam}	**1.45 [.49]**	**1.70 – 2.04**	--	--
μ_{AUDIT}	**1.86 [1.54]**	**1.40 – 1.51**	--	--
ϕ_{FF}	**.18 [.30]**	**.13 – .23**	**.18 [.02]**	**.14 – .22**
ϕ_{AA}	**.32 [.29]**	**.28 – .36**	**.31 [.02]**	**.28 – .35**
$\phi_{AUDIT \to Fam}$	.01 [.04]	-.02 – .04	.02 [.02]	-.02 – .05
$\phi_{Fam \to AUDIT}$	.00 [.03]	-.06 – .05	.00 [.02]	-.04 – .03
$\log(\pi_{Fam})$	**-1.39 [1.58]**	**-1.55 – -1.22**	**.84[.01]**	**.81 – .87**
$\log(\pi_{AUDIT})$	**-.89 [2.87]**	**-1.06 – -.51**	**.81 [.01]**	**.79 – .84**

Note. Unstandardized Point Estimates from Unconditional Models reflects the mean, standard deviation, and 95% credibility interval of the eight model parameters estimated in the unconditional model. Standardized estimates reflect the within-person associations of each of the eight parameters. 95% credibility intervals that do not contain zero are viewed as credible differences. Fam = LFQ family score, FF = LFQ Family autocorrelation, AA = AUDIT autocorrelation, $\phi_{Fam \to AUDIT}$ represents the cross-lagged regression, and $\log(\pi_{AUDIT})$ represents within-person variability.

© 2024 Sperry SH et al. *JAMA Network Open*

eTable 7. Unconditional DSEM model: LFQ Friend and AUDIT

	Unstandardized Point Estimates		Standardized Estimates	
**Variable**	**Mean [SD]**	**95% Credibility Interval**	**Estimate [Posterior S.D]**	**95% Credibility Interval**
μ_{Fnd}	**1.33 [.42]**	**1.28– 1.38**	--	--
μ_{AUDIT}	**1.85 [1.52]**	**1.70 – 2.03**	--	--
ϕ_{FF}	**.23 [.32]**	**.18 – .28**	**.22 [.02]**	**.18 – .27**
ϕ_{AA}	**.32 [.28]**	**.28 – .36**	**.32 [.02]**	**.29 – .35**
$\phi_{AUDIT\rightarrow Fnd}$	.02 [.03]	-.00 – .04	.04 [82.94]	.01 – .07
$\phi_{Fnd\rightarrow AUDIT}$	-.01 [.03]	-.05 – .05	-.01 [.02]	-.04 – .03
$\log(\pi_{Fnd})$	**-1.51 [1.56]**	**-1.67 – -1.35**	**.82 [***]**	**.79 – .85**
$\log(\pi_{AUDIT})$	**-.78 [2.86]**	**-1.04– -.50**	**.81 [.01]**	**.78 – .83**

Note. Unstandardized Point Estimates from Unconditional Models reflects the mean, standard deviation, and 95% credibility interval of the eight model parameters estimated in the unconditional model. Standardized estimates reflect the within-person associations of each of the eight parameters. 95% credibility intervals that do not contain zero are viewed as credible differences. Fnd = LFQ friend score, FF = LFQ Friend autocorrelation, AA = AUDIT autocorrelation, $\phi_{Fnd\rightarrow AUDIT}$ represents the cross-lagged regression, and $\log(\pi_{AUDIT})$ represents within-person variability.

© 2024 Sperry SH et al. *JAMA Network Open*

eTable 8. Unconditional DSEM model: LFQ Family and AUDIT

	Unstandardized Point Estimates		Standardized Estimates	
**Variable**	**Mean [SD]**	**95% Credibility Interval**	**Estimate [Posterior S.D]**	**95% Credibility Interval**
μ_{Home}	**0.92 [.63]**	**.86 – .99**	--	--
μ_{AUDIT}	**1.85 [1.53]**	**1.70 – 2.03**	--	--
ϕ_{HH}	**.21 [.27]**	**.17 – .25**	**.21 [.02]**	**.17 – .24**
ϕ_{AA}	**.32 [.29]**	**.28 – .36**	**.32 [.02]**	**.29 – .35**
$\phi_{AUDIT \rightarrow Home}$	.01 [.03]	-.00 – .03	.02 [.02]	-.01 – .05
$\phi_{Home \rightarrow AUDIT}$	-.05 [.03]	-.08 – .01	-.03 [.02]	-.05 – .01
$\log(\pi_{Home})$	**-1.86 [2.09]**	**-2.07 – -1.66**	**.85 [.01]**	**.83 – .88**
$\log(\pi_{AUDIT})$	**-.80 [2.87]**	**-1.07– -.55**	**.81 [.01]**	**.78 – .83**

Note. Unstandardized Point Estimates from Unconditional Models reflects the mean, standard deviation, and 95% credibility interval of the eight model parameters estimated in the unconditional model. Standardized estimates reflect the within-person associations of each of the eight parameters. 95% credibility intervals that do not contain zero are viewed as credible differences. Home = LFQ home score, HH = LFQ home autocorrelation, AA = AUDIT autocorrelation, $\phi_{Home \rightarrow AUDIT}$ represents the cross-lagged regression, and $\log(\pi_{AUDIT})$ represents within-person variability.

© 2024 Sperry SH et al. *JAMA Network Open*

eTable 9. Unconditional DSEM model: LFQ Work and AUDIT

Variable	Unstandardized Point Estimates		Standardized Estimates	
	Mean [SD]	95% Credibility Interval	Estimate [Posterior S.D]	95% Credibility Interval
μ_{Work}	**1.80 [.55]**	**1.74 – 1.86**	--	--
μ_{AUDIT}	**1.86 [1.53]**	**1.70 – 2.03**	--	--
ϕ_{WW}	**.20 [.28]**	**.16 – .25**	**.20 [.02]**	**.16 – .24**
ϕ_{AA}	**.32 [.29]**	**.28 – .36**	**.32 [.02]**	**.29 – .35**
$\phi_{AUDIT\rightarrow Work}$	**.02 [.03]**	**.00 – .04**	**.05 [.02]**	**.02 – .08**
$\phi_{Work\rightarrow AUDIT}$	-.01 [.03]	-.04 – .06	.00 [.02]	-.03 – .04
$\log(\pi_{Work})$	**-1.35 [1.32]**	**-1.48 – -1.21**	**.85 [.01]**	**.82 – .88**
$\log(\pi_{AUDIT})$	**-.78 [2.86]**	**-1.05– -.51**	**.81 [.01]**	**.79 – .84**

Note. Unstandardized Point Estimates from Unconditional Models reflects the mean, standard deviation, and 95% credibility interval of the eight model parameters estimated in the unconditional model. Standardized estimates reflect the within-person associations of each of the eight parameters. 95% credibility intervals that do not contain zero are viewed as credible differences. Work = LFQ work score, WW = LFQ work autocorrelation, AA = AUDIT autocorrelation, $\phi_{Work\rightarrow AUDIT}$ represents the cross-lagged regression, and $\log(\pi_{AUDIT})$ represents within-person variability.

© 2024 Sperry SH et al. *JAMA Network Open*

eFigure 3. Conditional DSEM model for GAD7 and AUDIT

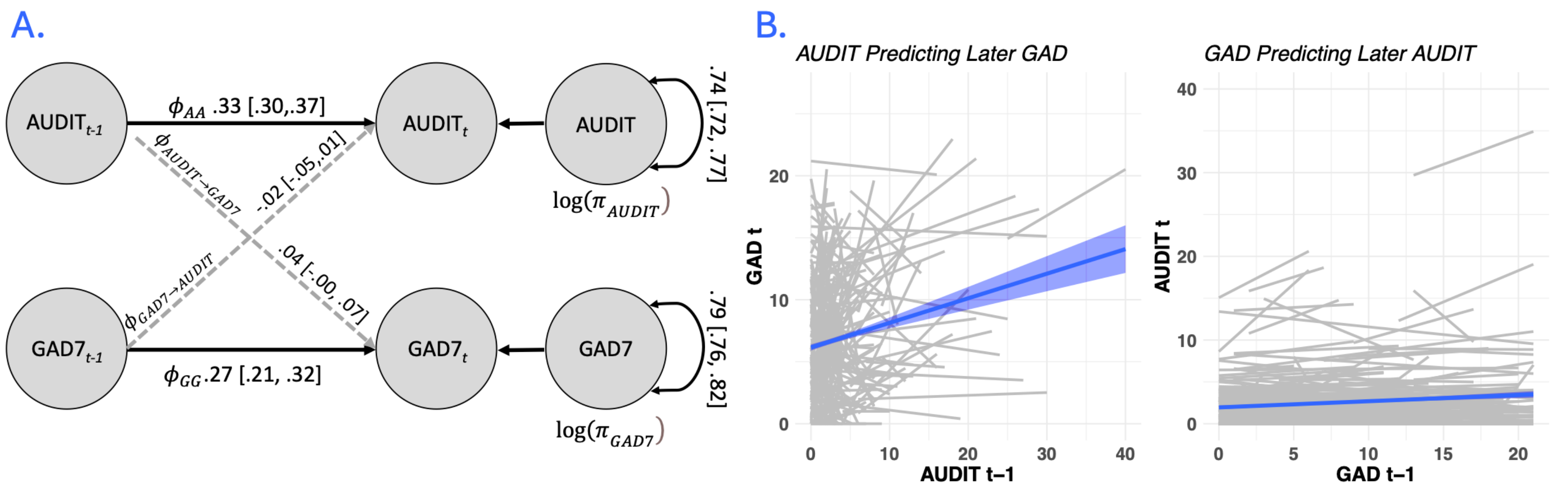

Note. For each parameter we present the standardized estimate followed by the 95% credibility interval in brackets. Dashed lines are not credible (95% credibility interval contains zero). Solid lines are viewed as credible differences (95% credibility interval does not include zero). t=time; t-1 = time – 1 observation; ϕ_{AA} = Autocorrelation of AUDIT; $\log(\pi_{AUDIT})$ = within-person variability in AUDIT. **Panel A.** This diagram represents the within-person parameters estimated in the conditional DSEM model with the GAD7 and AUDIT measures. ϕ_{GG} = Autocorrelation of GAD7; $\phi_{AUD \rightarrow GAD\&}$ = Cross-lagged relationship between AUDIT and GAD7 at the next timepoint; $\phi_{GAD7 \rightarrow AUDIT}$ = Cross-lagged relationship between GAD7 and AUDIT at the next timepoint; $\log(\pi_{GAD7})$ = Within-person variability of GAD7. **Panel B**. This graph is a plot of each person's cross-lagged regression between the AUDIT and GAD7 at the next

© 2024 Sperry SH et al. *JAMA Network Open*

timepoint (left) and the GAD7 and the AUDIT at the next timepoint (right). Each grey line represents an individual in the study. The blue solid line represents the group mean with the standard error around the mean.

© 2024 Sperry SH et al. *JAMA Network Open*

eFigure 4. Conditional DSEM Model for LFQ Family, Friend, and Home with AUDIT

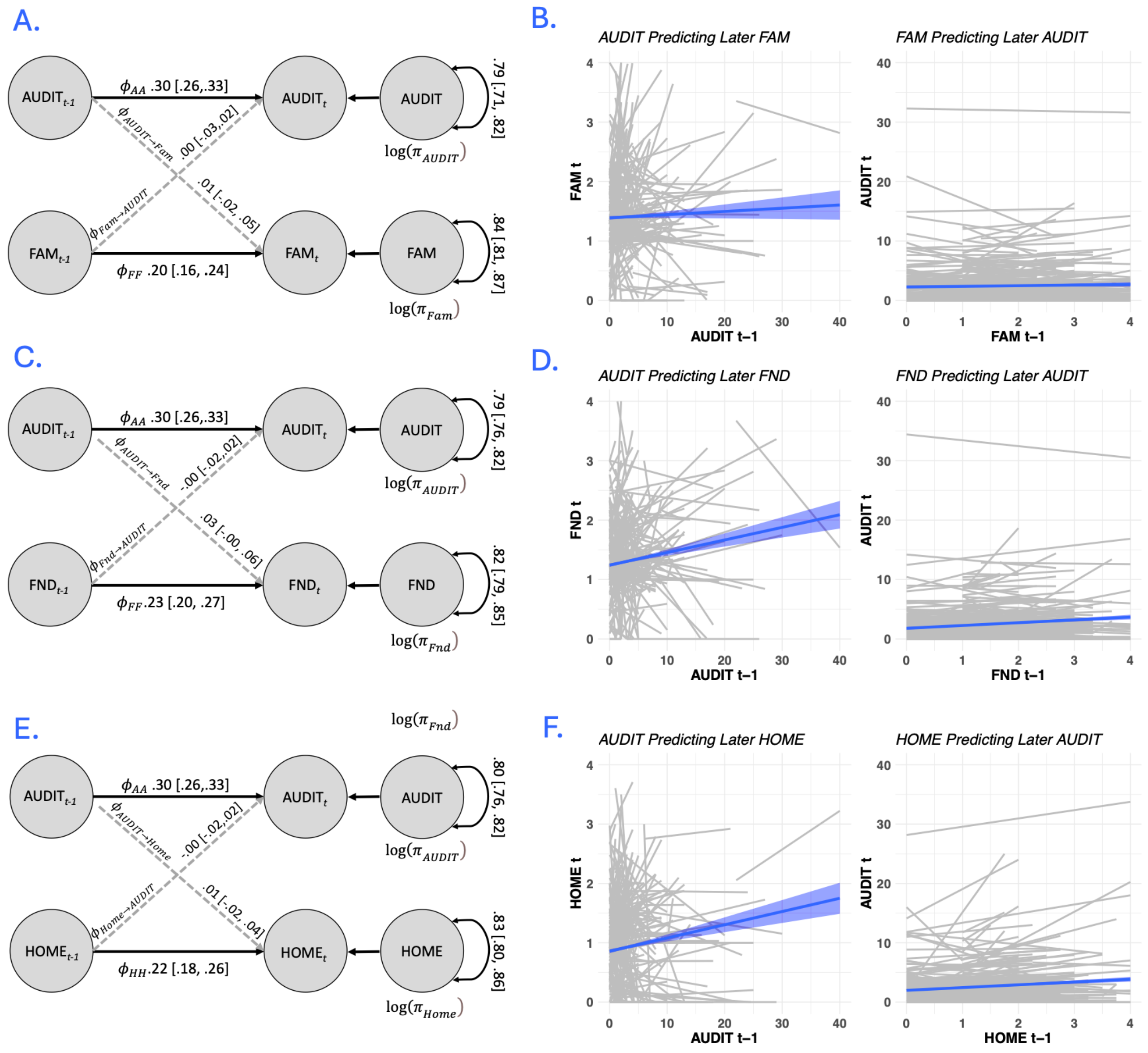

Note. For each parameter we present the standardized estimate followed by the 95% credibility interval in brackets. Dashed lines are not credible (95% credibility interval contains zero). Solid lines are viewed as credible differences (95% credibility interval does not include zero). t=time; t-1 = time – 1 observation; ϕ_{AA} = Autocorrelation of AUDIT; $\log(\pi_{AUDIT})$ = within-person variability in AUDIT. **Panel A.** This diagram represents the within-person parameters estimated

in the conditional DSEM model with the LFQ family and AUDIT measures. ϕ_{FF} = Autocorrelation of LFQ family; $\phi_{AUDIT \to Fam}$ = Cross-lagged relationship between AUDIT and LFQ fam at the next timepoint; $\phi_{Fam \to AUDIT}$ = Cross-lagged relationship between LFQ family and AUDIT at the next timepoint; $\log(\pi_{Fam})$ = Within-person variability of LFQ family. **Panel B**. This graph is a plot of each person's cross-lagged regression between the AUDIT and LFQ family at the next timepoint (left) and the LFQ family and the AUDIT at the next timepoint (right). Each grey line represents an individual in the study. The blue solid line represents the group mean with the standard error around the mean. **Panel C.** This diagram represents the within-person parameters estimated in the conditional DSEM model with the LFQ friend and AUDIT measures. ϕ_{FF} = Autocorrelation of LFQ friend; $\phi_{AUDIT \to Fnd}$ = Cross-lagged relationship between AUDIT and LFQ friend at the next timepoint; $\phi_{Fnd \to AUDIT}$ = Cross-lagged relationship between LFQ friend and AUDIT at the next timepoint; $\log(\pi_{Fnd})$ = Within-person variability of LFQ friend. **Panel D**. This graph is a plot of each person's cross-lagged regression between the AUDIT and LFQ friend at the next timepoint (left) and the LFQ friend and the AUDIT at the next timepoint (right). Each grey line represents an individual in the study. The blue solid line represents the group mean with the standard error around the mean. **Panel E.** This diagram represents the within-person parameters estimated in the conditional DSEM model with the LFQ home and AUDIT measures. ϕ_{HH} = Autocorrelation of LFQ homed; $\phi_{AUDIT \to Home}$ = Cross-lagged relationship between AUDIT and LFQ home at the next timepoint; $\phi_{Home \to AUDIT}$ = Cross-lagged relationship between LFQ home and AUDIT at the next timepoint; $\log(\pi_{Home})$ = Within-person variability of LFQ friend. **Panel F**. This graph is a plot of each person's cross-lagged regression between the AUDIT and LFQ home at the next timepoint (left) and the LFQ home and the AUDIT at the next timepoint

© 2024 Sperry SH et al. *JAMA Network Open*

(right). Each grey line represents an individual in the study. The blue solid line represents the group mean with the standard error around the mean.

© 2024 Sperry SH et al. *JAMA Network Open*
