# Supplementary material for: Longitudinal Interplay Between Alcohol Use, Mood, and Functioning in Bipolar Spectrum Disorders
Source: JAMA Netw Open. 2024 Jun 7;7(6):e2415295. doi: 10.1001/jamanetworkopen.2024.15295 (PMC11161848; doi:10.1001/jamanetworkopen.2024.15295)
Supplement: Supplement 2. — Data Sharing Statement [file jamanetwopen-e2415295-s002.pdf]

Data Sharing Statement

Sperry. Longitudinal Interplay Between Alcohol Use, Mood, and Functioning in Bipolar Spectrum Disorders. *JAMA Netw Open*. Published June 07, 2024. doi:10.1001/jamanetworkopen.2024.15295

Data
Data available: No

Additional Information
Explanation for why data not available: Longitudinal and outcomes data used in the present study, along with data dictionaries, are available subject to review of the proposed analyses and acceptance of a Data Use Agreement. All PLS-BD data and samples are available through the Heinz C. Prechter Genetic Repository, distributed by the University of Michigan Central Biorepository (CBR). Enquiries can be addressed at http://www.prechterprogram.org/data. Data dictionaries and supporting documentation (e.g., statistical/analytic code) is available upon request from Dr. Sarah Sperry (sperrys@umich.edu).
